# Predicting seizure recurrence after an initial seizure-like episode from routine clinical notes using large language models: a retrospective cohort study

**DOI:** 10.1016/S2589-7500(23)00179-6

**Published:** 2023-12

**Authors:** Brett K Beaulieu-Jones, Mauricio F Villamar, Phil Scordis, Ana Paula Bartmann, Waqar Ali, Benjamin D Wissel, Emily Alsentzer, Johann de Jong, Arijit Patra, Isaac Kohane

**Affiliations:** Department of Medicine, University of Chicago, Chicago, IL, USA (B K Beaulieu-Jones PhD); Department of Biomedical Informatics, Harvard Medical School, Boston, MA, USA (B K Beaulieu-Jones, E Alsentzer PhD, Prof I Kohane MD PhD); Department of Neurology,The Warren Alpert Medical School of Brown University, Providence, RI, USA (M F Villamar MD); UCB, Brussels, Belgium (P Scordis PhD, A P Bartmann MD PhD, W Ali PhD, A Patra PhD); Division of Biomedical Informatics, Cincinnati Children’s Hospital Medical Center, Cincinnati, OH, USA (B D Wissel MD PhD); UCB Biosciences, Monheim, Germany (J de Jong PhD)

## Abstract

**Background:**

The evaluation and management of first-time seizure-like events in children can be difficult because these episodes are not always directly observed and might be epileptic seizures or other conditions (seizure mimics). We aimed to evaluate whether machine learning models using real-world data could predict seizure recurrence after an initial seizure-like event.

**Methods:**

This retrospective cohort study compared models trained and evaluated on two separate datasets between Jan 1, 2010, and Jan 1, 2020: electronic medical records (EMRs) at Boston Children’s Hospital and de-identified, patient-level, administrative claims data from the IBM MarketScan research database. The study population comprised patients with an initial diagnosis of either epilepsy or convulsions before the age of 21 years, based on International Classification of Diseases, Clinical Modification (ICD-CM) codes. We compared machine learning-based predictive modelling using structured data (logistic regression and XGBoost) with emerging techniques in natural language processing by use of large language models.

**Findings:**

The primary cohort comprised 14 021 patients at Boston Children’s Hospital matching inclusion criteria with an initial seizure-like event and the comparison cohort comprised 15 062 patients within the IBM MarketScan research database. Seizure recurrence based on a composite expert-derived definition occurred in 57% of patients at Boston Children’s Hospital and 63% of patients within IBM MarketScan. Large language models with additional domain-specific and location-specific pre-training on patients excluded from the study (F1-score 0·826 [95% CI 0·817–0·835], AUC 0·897 [95% CI 0·875–0·913]) performed best. All large language models, including the base model without additional pre-training (F1-score 0·739 [95% CI 0·738–0·741], AUROC 0·846 [95% CI 0·826–0·861]) outperformed models trained with structured data. With structured data only, XGBoost outperformed logistic regression and XGBoost models trained with the Boston Children’s Hospital EMR (logistic regression: F1-score 0·650 [95% CI 0·643–0·657], AUC 0·694 [95% CI 0·685–0·705], XGBoost: F1-score 0·679 [0·676–0·683], AUC 0·725 [0·717–0·734]) performed similarly to models trained on the IBM MarketScan database (logistic regression: F1-score 0·596 [0·590–0·601], AUC 0·670 [0·664–0·675], XGBoost: F1-score 0·678 [0·668–0·687], AUC 0·710 [0·703–0·714]).

**Interpretation:**

Physician’s clinical notes about an initial seizure-like event include substantial signals for prediction of seizure recurrence, and additional domain-specific and location-specific pre-training can significantly improve the performance of clinical large language models, even for specialised cohorts.

**Funding:**

UCB, National Institute of Neurological Disorders and Stroke (US National Institutes of Health).

## Introduction

The evaluation and management of first-time seizure-like events in children poses considerable challenges. Although some of these episodes might represent epileptic seizures, seizure mimics are also common in paediatric populations.^1,2^ Physicians might not directly observe the initial seizure, or even know whether a seizure has occurred, and so they define seizure-like to include any initial visit with an International Classification of Diseases (ICD) code for epilepsy, seizures (including febrile), and convulsions (appendix p 1). When a child presents with a first-time transient neurological episode, a thorough clinical evaluation is necessary to distinguish between an epileptic seizure and seizure mimic. Even when, after expert evaluation, an episode is considered to correspond to an epileptic seizure, risk stratification can be difficult in clinical practice.^3^ In many children, a first-time seizure could represent a singular event, sometimes precipitated by an acute condition, with a low risk of recurrence. In other cases, this event might be the first of a recurring series of seizures, which will eventually lead to an established diagnosis of epilepsy.

Accurately predicting seizure recurrence is important for both the diagnosis of epilepsy and the treatment of seizures. A patient is diagnosed with epilepsy if they meet any of the following criteria: one unprovoked or reflex seizure with a high risk of recurrence (typically ≥60%), at least two unprovoked or reflex seizures occurring more than 24 h apart, or the clinical identification of a specific epilepsy syndrome.^4,5^ The diagnosis of epilepsy is correlated with but not necessarily linked to the decision to start antiseizure medications (ASMs). The decision to start an ASM is typically based on clinical suspicion of epileptic seizures, findings from ancillary neurodiagnostic studies, and the estimated risk of seizure recurrence.^6–8^ With some exceptions, children are typically not started on ASMs after the first unprovoked seizure.^9–14^ When a child has a second unprovoked seizure they are diagnosed with epilepsy and this generally, but not always, indicates a sufficiently high risk of seizure recurrence to justify initiation of an ASM.^15^ However, certain epilepsy syndromes in children have a relatively favourable long-term prognosis and might not require chronic ASM therapy.^4^ Therefore, the decision to start an ASM is based on the risk of recurrent seizures, but must also consider the relative risk reduction an ASM could provide, combined with the risks associated with long-term use of ASMs (eg, endocrine dysfunction and bone disease). The choice of ASM should therefore be guided by epilepsy type, patient comorbidities, cost, side-effect profile, and the potential for other favourable clinical effects of the ASM.^16^

Previous studies have estimated that when a first, unprovoked, afebrile seizure occurs in a child with no previous history of neurological illness and a normal neurological examination, the recurrence rate for additional afebrile unprovoked seizures is approximately 25% within 1 year and 45–50% within 3 years.^3,8,17^ Most studies predicting seizure recurrence have focused on whether seizures will recur after the withdrawal of ASMs or on predicting outcomes following epilepsy surgery.^18–26^ Studies predicting seizure recurrence after an initial seizure have largely been focused on specific aetiologies^27^ or purpose-collected data (including manual chart review)—for example, by recruiting patients to registries or recording specific information in seizure databases.^14,28,29^ Kim and colleagues^30^ developed a model with data from the Multicentre trial for Early Epilepsy and Single Seizures (MESS) that categorised the risk of seizure recurrence as low (<30%), medium (30–49%), or high (≥50%). To date, MESS is the largest study of people with single seizures and early epilepsy. Kim and colleagues^30^ concluded that there might be a benefit of initiating treatment in patients with a medium or high risk of recurrence. However, this model had poor discrimination for the MESS database (C-statistic 0·59) and the three external databases it was tested in (C-statistic: 0·55–0·60).^31^ van Diessen and colleagues^32^ developed a logistic regression model in a cohort of 451 children (with external validation carried out in a cohort of 187 children) that predicted the risk of seizure recurrence on the basis of clinical and electroencephalogram (EEG) characteristics (AUC=0·86). To the best of our knowledge, no previous models have used raw data from electronic medical records (EMRs) collected for routine care, or directly input clinical notes from EMRs, for the purpose of predicting the risk of seizure recurrence.

A recent trend in natural language processing (NLP) based on deep learning techniques involves self-supervised learning over massive unlabelled general textual datasets to capture the intrinsic structure of the data, and subsequent application of these large pre-trained models to a specific problem domain by fine-tuning, usually on a much smaller dataset.^33^ These pre-trained models have been shown to outperform heavily engineered, task-specific architectures on many sentence-level and token-level tasks.^34–36^ Jiang and colleagues^37^ trained a large language model for general tasks such as re-admission, in-hospital mortality, length of stay, and comorbidity index prediction. Although direct comparisons between different cohorts are challenging (eg, demographic differences and rates of events), this model achieved similar performance to previous NLP-based approaches and logistic regression approaches based on expert-derived features.^38^ Previous work has shown that performance close to this level can be achieved by predicting solely from similar patient outcomes (eg, in-hospital mortality and re-admission rates),^39^ suggesting that prediction models based on EMRs might not derive their signal purely from objective measures of patient state, but are also likely to benefit from physicians’ beliefs and decision making.

The actions taken and notes captured in EMRs depict some of the thoughts and prognostications of physicians.^39^ Clinical orders and even the timing of these orders have been shown to hold substantial information about patient risk. For example, Agniel and colleages^40^ observed that the timing of a white blood cell count was more predictive of 3-year survival than the actual results from the laboratory. They observed that for 68% of 118 tests, process information (eg, timing) was more informative than pathophysiology. Predictions based on physician-collected data could therefore provide a strong baseline for future models designed to predict recurrence risk directly from patient measurements and diagnostics such as EEG, head CTs, and brain MRIs. Surpassing this baseline will be an important measure indicating whether future models can help physicians beyond their current intuition.

The goal of this study was to ascertain whether naturally collected data in EMRs (ie, data collected as part of routine care and not specifically for the purpose of predicting future seizures) contain signals predictive of the risk of seizure recurrence. We performed this ascertainment by training machine learning models to predict the risk of seizure recurrence in a patient with an initial seizure-like event. Given that it is not easy for humans to translate their intuition to exact percentages (eg, to specify whether a particular patient has a ≥60% chance of seizure recurrence^5^), we aimed to evaluate whether machine learning methods could quantify the risk of seizure recurrence from information naturally recorded by physicians.^41^

We compared traditional approaches (logistic regression and XGBoost^42^) applied to structured EMR data with emerging approaches for NLP to predict whether a patient presenting with seizure-like events will have additional seizures within the next 2 years. We relied primarily on a single paediatric health system’s EMRs but leveraged data from a nationwide administrative claims dataset to explore how our findings might differ from the broader population, including non-tertiary care settings.

## Methods

### Data sources

This retrospective cohort study analysed data from two separate populations and two different data modalities: EMRs from Boston Children’s Hospital and administrative claims data from the IBM MarketScan dataset. We included IBM MarketScan data to enable comparison of a paediatric tertiary care facility (Boston Children’s Hospital) with general nationwide data, in order to understand potential differences based on data modality, population source, and care setting. Comparisons between datasets were made with parametric methods where normality was observed (two-sample *t*-test) and non-parametric methods (Mann-Whitney *U* test) where normality could not be discerned.

Using i2b2 (software for patient cohort identification),^43^ we identified 14 021 patients meeting the inclusion and outcome criteria (provided below) at Boston Children’s Hospital between Jan 1, 2010, and Jan 1, 2020 (panel^44,45^). We extracted data on patient demographics, diagnoses, medications (home and in-hospital), procedures, and laboratory and clinical notes from the i2b2 data model for analyses. In addition to the structured medication data captured discretely in the EMR medication orders, administration, and history, we extracted medication data from the clinical notes at Boston Children’s Hospital, because we found that patients frequently had mentions of external prescriptions in their notes that were not captured in the structured data. We extracted medication data by first identifying appropriate sections with a rules-based approach to identify sections of clinical notes referencing medications (history, changes, orders, and discharge medication lists). We used SciSpacy^46^ for negation detection and to link medications to RxNorm^47^ so that medications could be categorised by drug class. We did a manual chart review of 250 clinical notes in which extracted medications were identified and 250 notes in which no medications were identified. In this process we found two errors: a misspelling and an abbreviation (levetiracetam referred to as “LEV”). In both cases, other notes for the patient in question identified and associated the medication with the patient.

We used de-identified, patient-level, administrative claims data from the IBM MarketScan research database.^48^ The IBM MarketScan research database includes a broad population based on claims after payment and adjudication. It includes covered periods for more than 273 million individuals with both private (approximately 50%) and public insurance (eg, Medicaid and the Children’s Health Insurance Program [CHIP]; approximately 50%). The data provided for this study included patient demographics and medical and prescription claims (inpatient and outpatient encounters, medications, diagnoses, procedures, and laboratory tests ordered). Within the IBM MarketScan research database, 15 062 patients matched the inclusion criteria.

### Patient inclusion criteria

The study population comprised patients with an initial diagnosis of either epilepsy or convulsions before the age of 21 years, based on International Classification of Diseases, Clinical Modification codes (ICD9-CM and ICD10-CM), with sufficient data to determine whether it was likely to be a first-time event and for follow-up outcome assessment.

Patients were required to fulfil three criteria. First, they had to have an encounter (at Boston Children’s Hospital) or insurance coverage (in IBM MarketScan) for any reason at least 1 year before an initial seizure-like episode to enrich for those patients without a history of seizures (patients having a first seizure-like event before 1 year are required to have an encounter for other purposes at least 1 week before the index encounter to filter for patients being referred after having previous seizures). Second, they were required to have an index encounter for epilepsy or convulsions (appendix p 1). Notably, the definition for this index encounter is broader than the definitions used to identify outcomes because the code used at the time of initial diagnosis might differ from that used in future visits for the same issue or even the clinical notes from the initial visit. Third, they were required to have an additional encounter for any purpose (at Boston Children’s Hospital) or coverage (in IBM MarketScan) at least 2 years after the index event.

The goal of this definition was to enrich the cohort for patients having their first seizure-like event by including a sufficiently long period before the index date, while also having sufficient follow-up to evaluate whether seizures recurred. Unlike other large database studies defining epilepsy,^49–51^ we sought to identify patients with an initial seizure or seizure-like event. Therefore, it did not seem appropriate to use prescription release encounters to define the initial cohort.

Notably, any definitions used to define these cohorts will have their limitations. In this case, the requirement of any encounter at least 1 year earlier excluded patients who were healthy and did not receive care at Boston Children’s Hospital. The alternative, however, was risking the inclusion of patients who received their care outside of Boston Children’s Hospital, had a series of seizures, and then arrived at Boston Children’s Hospital for the first time. These patients would not be representative of those having an initial seizure-like episode. Additionally, the requirement for an encounter at least 2 years after the index event was necessary to have a sufficient follow-up time to observe outcomes. It also helped to filter patients who were seen at Boston Children’s Hospital as a one-off specialty referral for a second opinion, as these patients generally would not meet the first and third criteria.

### Outcome criteria

Because neither the EMR nor claims data will capture all seizures occurring in these two cohorts, we developed meaningful definitions of seizure recurrence. We developed a binary composite label to serve as a proxy for a patient having an additional seizure after the first event (panel). To define this label we used measures of epilepsy available in real-world data, including adjunctive therapy, diagnoses of status epilepticus after initial presentation, seizure-related inpatient admissions, seizure-related health-care utilisation, all health-care utilisation, seizure-related diagnostic testing, and seizure-related procedures performed (definitions provided in the appendix p 4). Outcome definitions were in part derived from previous work validating coding definitions for administrative claims data.^52^ Patients meeting any of the criteria outlined in the panel were considered to be positive for seizure recurrence and only those not matching any criteria were considered to be seizure-free. This is an imperfect proxy definition due to the absence of specific data capture around seizures, but it incorporates substantial expert knowledge.

The definition for outcome epilepsy-specific ICD billing codes (ICD-9 code 345.** or ICD-10 code G40.*, eg, excluding convulsions ICD9–780.** and ICD10-R56.*) has been shown to have high sensitivity and specificity for epilepsy.^51^ The use of ICD-based definitions allows for cross-comparison between EMR and claims data (EMR-only definitions might be more accurate but require additional validation and cannot be used with claims data). For procedures, we required both an epilepsy-related procedure code in an encounter with an epilepsy-specific diagnosis (as defined above) as the primary or admitting code. We also required another epilepsy-specific diagnosis at least 1 week after the procedure to avoid the issue of procedures being misclassified as recurrence, as reported in the literature.^53^ The included medications are provided in the appendix (pp 2–3), including both first-line epilepsy treatments and ASMs indicated for adjunctive use.

We required status epilepticus to either be the primary or admitting code, or for the primary or admitting code to be one of the epilepsy-specific ICD billing codes. This approach filtered for cases of febrile status epilepticus. Since coding is not perfect, we determined that febrile status epilepticus coded as afebrile status epilepticus is reasonable to include in the status epilepticus category, because febrile status epilepticus might occur in the context of Dravet syndrome or in entities such as febrile infection-related epilepsy syndrome (FIRES). FIRES is a subcategory of new-onset refractory status epilepticus, which is associated with substantial morbidity and mortality.^54^ The consensus definition of FIRES has been published,^54^ and there is no specific ICD-10 code for FIRES. Since status epilepticus is a neurological emergency, we aimed to identify any cases of status epilepticus regardless of whether they were febrile or not. Additionally, although ASMs in general are not recommended for recurrent febrile seizures, which are assumed to be benign, we believe our outcome definition aligns with the fact that for some patients, complex febrile seizures or febrile status epilepticus might be characteristic of an underlying epilepsy syndrome, and might therefore indicate an increased risk of afebrile seizures.^55,56^ As such, we did not include specific codes for febrile seizures in the outcome definition for recurrence, but acknowledge that if a patient is treated with ASMs (ie, the physician believes the patient is high risk) or has status epilepticus, they will be included in the recurrent group.

### Prediction of seizure outcomes

We performed five-fold cross-validation using 80% of the dataset for training (11 216 patients at Boston Children’s Hospital and 12 049 within IBM MarketScan) and 20% for evaluation (2805 patients at Boston Children’s Hospital and 3013 within IBM MarketScan). We repeated this five-fold cross-validation for each individual outcome described in the panel as well as the composite score (ie, meeting any individual outcome) to assess the stability of predictive performance across potential outcome measures. Given the sample sizes and because the purpose of this study was to understand whether clinical data sources encode information about seizure recurrence after an initial seizure-like event, rather than deployment, we did not do additional hyperparameter tuning for the clinical longformer models and therefore only used a train and test split of data. We calculated 95% CIs with bootstrapping to 1000 random samples.

We performed predictions of seizure recurrence using XGBoost^42^ and the scikit-learn implementation of logistic regression^57^ as a baseline comparison. We performed an extensive hyperparameter sweep for both logistic regression and XGBoost (>600 000 total parameter combinations). Full details are available in the appendix (p 5). We examined feature sets including diagnoses in either ICD or PheCode form, procedures (Current Procedural Terminology [CPT] and ICD), medications (National Drug Codes [NDCs] rolled up with RxNorm), and laboratory results (logical observation identifiers names and codes [LOINCs]). Pipelines with both classifiers showed the strongest performance with the full feature set features where diagnoses were represented by PheCodes.

We first constructed a feature vector containing the counts of each observed code in the year before the initial seizure diagnosis (up to and including the day of the first seizure diagnosis).^58^ Each feature vector had a corresponding set of labels, but the parameter sweep and primary evaluations were done with the composite score as a binary label.

We then performed normalisation of these vectors, and found that for both pipelines the strongest performing normalisation technique was to represent a patient as a vector of the counts of occurrences for each code and then perform log-normalisation: log(count occurrences + 1), clipped to a maximum of 1. This approach allows contribution of multiple diagnoses for patients while avoiding large outliers. Each subsequent diagnosis decays under the assumption that the first diagnosis is most informative, followed by the second, and so on. Differences between, for example, eight and nine diagnoses might be more likely to be driven by health-care dynamics and billing processes.

After all features were normalised, feature selection techniques were evaluated on the training dataset, based on frequency, information content, and association with outcomes (recursive feature elimination and χ2 statistic ranking). We found that including all features present in at least 1% of the data was best for XGBoost (2115 features) and selecting the top 500 best features according to the χ2 test statistic was best for logistic regression.

Finally, we evaluated parameters for the classifiers themselves. For many parameter settings, it was not possible to determine that one model significantly outperformed another. In these cases, the simpler (eg, fewer estimators, reduced depth, or fewer included features) and default parameters were used. For logistic regression, we identified that the default parameters for logistic regression with “elasticnet” penalisation with class balancing performed as well as any other included parameter set (no parameters showed a significant cross-validation score). For XGBoost, we found non-inferior performance with the default parameters except when setting the number of estimators to 500 and setting lambda and alpha regularisation terms to 0·1.

We acknowledge this parameter sweep has the potential to inflate performance due to multiple testing. Because the structured EMR data are only meant for baseline comparison, we chose the strongest baseline possible to compare with the NLP-based models. This approach reduces the potential for an under-optimised baseline comparison. Details of the full pipeline parameter sweeps of initial feature sets, normalisation, feature selection, and classifier parameters are available in the appendix (p 5).

Long-sequence transformers^40^ were proposed with the idea of sparse attention mechanism for more efficient scaling with sequence length, thus enhancing the ability of modelling long-term dependency. Here, we used the clinical-longformer model^42^ as the base for our experi-ments, which is pre-trained on an unrelated clinical corpus (MIMIC-III clinical notes^43^).

We performed additional pre-training on clinical notes from Boston Children’s Hospital to create the Boston Children’s Hospital clinical-longformer model. To do this, we selected the relevant notes, defined as all neurology notes, communications (eg, patient messages), imaging and EEG reports (eg, brain MRI and head CT scans), and discharge summaries from patients who had diagnoses for epilepsy or convulsions but did not match our inclusion criteria (eg, had a seizure on their first visit, did not have follow-up data, and so on).

These criteria provided 8 549 848 clinical notes for pre-training (1·7 billion tokens), several times more tokens than the clinical pre-training of the base model with MIMIC (approximately 2 million notes). Less than 5% of around 8·5 million notes were longer than 4096 tokens. Notes longer than 4096 tokens were split into multiple notes with an overlapping window of 512 tokens. We used Nvidia RTX 8000 with 48 GB of RAM. This allowed for a per-device batch size of 4 and, by accumulating the gradient over two steps, an effective batch size of 32. All other hyperparameters were kept the same as the original clinical-longformer model.^33^ We used the Hugging Face transformers library^59^ to run additional pre-training for 300 000 steps, taking approximately 1 week. We compared the model with additional or domain-specific pre-training to the base clinical-longformer model.

We first empirically evaluated the masked language modelling performance of the base clinical-longformer model and the Boston Children’s Hospital clinical-longformer model by providing prompts that include a missing word for the models to fill in (table 1). This approach allowed for an intuitive, qualitative comparison between the two models. We then fine-tuned each model on a classification prediction task using the relevant notes from patients who matched the inclusion criteria for the set of outcomes described in the panel. Fine-tuning was done with each note, predicting whether the patient would have seizure recurrence, yielding multiple notes per patient. We fine-tuned for up to five epochs, with early stopping based on training loss after every ten steps and a patience of ten (ie, 100 total steps). During fine-tuning we used a per-device batch size of 32 without gradient accumulation, for an effective batch size of 128. Additionally, layer-wise weight decay was set to 0·01. We performed fine-tuning using two approaches: where the weights of the entire model were trainable and where only the weights of the head of the model were trainable.

The predictions at the note level were aggregated to the patient level by selecting the class of the prediction with largest margin from the threshold (0·5). This aggregation can be interpreted as pseudo-confidence scores where the note with the most confident prediction in either direction was used. We explicitly did not perform an additional hyperparameter sweep for the clinical-longformer or Boston Children’s Hospital clinical-longformer fine-tuning, classification, or note aggregation steps, to avoid the risk of multiple testing issues during cross-validation. This aggregation strategy was chosen because it is intuitive and values notes encoding information leading to “confident” predictions. There are likely to be more sophisticated aggregation strategies that could improve performance but these strategies should be created and evaluated in a larger sample with additional levels of hold-out validation sets.

We did an ablation study over notes to understand where the predictive signal is encoded. We concretely trained separate Boston Children’s Hospital clinical-longformer models with notes from each note type and compared the performance of each model. The following note types were considered: neurology notes and communications (eg, messages to but not from patients, and messages describing interactions with patients), EEG reports, brain MRI reports, head CT reports, discharge summaries, and all relevant notes (all specified note types).

### Standard protocol approvals, registrations, and patient consent

The institutional review boards of Harvard Medical School and Boston Children’s Hospital reviewed and approved the research protocol and waived the requirement for written consent as the study presents no more than minimal risk to the privacy of individuals, uses only data collected as part of routine clinical care, and presents only aggregate results (which do not include any identifiable information), and the study could not practically be conducted without the waiver of informed consent.

### Role of the funding source

The study funders did not play a role in data collection, data analysis, or data interpretation, writing of the manuscript or the decision to submit the manuscript for publication.

## Results

The primary cohort comprised 14 021 children at Boston Children’s Hospital and the comparison cohort comprised 15 062 children within the IBM MarketScan research database (table 2). The included population had a non-epilepsy encounter (at Boston Children’s Hospital) or insurance coverage (in IBM MarketScan) at least 1 year before the index seizure-like event encounter (occurring between birth and 21 years of age, as defined in the appendix p 1). Additionally, as defined above, patients were required to have any encounter (at Boston Children’s Hospital) or insurance coverage (in IBM MarketScan) at least 2 years after the index encounter.

The two populations had a few key differences. Patients at Boston Children’s Hospital had, on average, an earlier onset of epilepsy than those in the IBM MarketScan dataset (6·76 *vs* 10·20 years; p<0·0001) and less focal epilepsy after an initial seizure-like event (50·9% *vs* 55·7%; p<0·0001). Information on race or ethnicity was not available in IBM MarketScan. Within Boston Children’s Hospital, groups of fewer than 30 patients (American Indian or Alaska Native, Native Hawaiian or Other Pacific Islander) were included in the “Other” category to avoid the potential for re-identification or identification of an individual being included in this study. Location information within IBM MarketScan was available only at the regional level. The requirement for non-seizure-like encounters enriched for patients located close to Boston Children’s Hospital who might be more likely to receive primary or other care at Boston Children’s Hospital. Within the cohort, 87·0% of patients were from Massachusetts and 96·0% were from New England (ie, from the states of Maine, Vermont, New Hampshire, Massachusetts, Connecticut, and Rhode Island). When removing the requirement for a non-seizure-like encounter at least 1 year before epilepsy, the proportion of patients within Massachusetts dropped to 70·1%, with 83·2% from New England (appendix p 10). This filter is important because patients outside the New England region might be more likely to be referrals and the first observed seizure-like event at Boston Children’s Hospital is unlikely to be the first event occurring in such patients.

To allow for comparison with the published literature, we compared initial treatment choices for those prescribed an ASM (appendix p 2). In line with the published literature, levetiracetam was the most common initial treatment. However, there was heterogeneity in treatment received, both in terms of the ASMs prescribed and the population of patients who received ASMs as rescue therapies or adjunctive therapy. Importantly, we observed that a lower proportion of patients at Boston Children’s Hospital (59·4%) had an indicated treatment present in their EMR during the study period than those observed in IBM MarketScan (74·7%; appendix p 7). This difference was reduced but not eliminated when performing NLP-based extraction of ASMs. Notably, ASMs have alternative indications. For example, in the paediatric population, gabapentin is prescribed far more frequently for management of neuropathic pain than for epilepsy.^59^ Although we required that the ASM appears in an encounter with an epilepsy diagnosis, it is likely that not all ASMs used for non-epilepsy indications were filtered out. The observed frequencies of gabapentin as a first-line treatment were 1·94% in IBM MarketScan, 2·88% in the structured Boston Children’s Hospital dataset, and 2·99% in the NLP-based Boston Children’s Hospital dataset (pp 7–8). At Boston Children’s Hospital we observed that patients receiving gabapentin as the first ASM after a seizure-like event were more likely to have focal epilepsy (67·8% *vs* 50·9%, p<0·0001), and had significantly higher rates of neuropathy, anxiety, pain, migraines, headaches, depression, infantile cerebral palsy, and encephalopathy (appendix p 8). These attributes might explain the higher rates of gabapentin use as a first-line treatment.

Table 3 shows epilepsy-related outcomes within 2 years of an initial seizure-like episode. The composite score is described in the Methods section as well as in the panel. Overall, the mean composite score for epilepsy severity was slightly higher (0·63) within the IBM MarketScan cohort than in the Boston Children’s Hospital cohort (0·57). The use of a composite score allowed for important comparisons between a specialised academic health system and a general nationwide cohort. This analysis illustrates the potential effects of biases that could be present given the nature of Boston Children’s Hospital (eg, referrals to a specialised academic health system might constitute more severe cases, and patients might receive follow-up care outside of Boston Children’s Hospital and therefore not be observed). These challenges provide an important context to results that are based on a single health system. Patients in the Boston Children’s Hospital cohort had fewer distinct encounter days with an epilepsy ICD code, and fewer encounters for status epilepticus. However, a higher proportion of encounters in the Boston Children’s Hospital cohort were related to inpatient admissions.

We evaluated the ability of various machine learning approaches to predict outcomes related to seizure recurrence, with particular attention paid to the composite score (figure A, B). Results are available in tabular form with 95% CIs in the appendix (p 7). We compared methods based on structured data (XGBoost and logistic regression) across both Boston Children’s Hospital and IBM MarketScan datasets. Within Boston Children’s Hospital we also compared two models, the base clinical-longformer model and the clinical-longformer model with additional pre-training with Boston Children’s Hospital data under two settings: allowing only the classifier layer to be trained during fine-tuning and allowing the entire transformer to be trained during fine-tuning.

Large language models with additional domain and location specific pre-training on patients excluded from the study (F1-score 0·826 [95% CI 0·817–0·835], AUC 0·897 [95% CI 0·875–0·913]) performed best. All large language models, including the base model without additional pre-training (F1-score 0·739 [95% CI 0·738–0·741], AUROC 0·846 [95% CI 0·826–0·861]), outperformed models trained with structured data. With structured data only, XGBoost outperformed logistic regression and XGBoost models trained with the Boston Children’s Hospital EMR (logistic regression: F1-score 0·650 [95% CI 0·643–0·657], AUC 0·694 [95% CI 0·685–0·705], XGBoost: F1-score 0·679 [0·676–0·683], AUC 0·725 [0·717–0·734]) performed similarly to models trained on the IBM MarketScan database (logistic regression: F1-score 0·596 [0·590–0·601], AUC 0·670 [0·664–0·675], XGBoost: F1-score 0·678 [0·668–0·687], AUC 0·710 [0·703–0·714]).

We observed several important trends: there was a significant improvement in performance for full transformer models over traditional approaches with the tabular structured data; it was important to allow the entire transformer model to be trainable when performing fine-tuning; and fine-tuning on an excluded cohort at Boston Children’s Hospital (the clinical-longformer model) provided a significant performance improvement over the base clinical-longformer model.

Additionally, transformer models pre-trained and fine-tuned on all relevant clinical notes (neurology notes, discharge summaries, imaging reports, and EEG reports) performed better than models pre-trained and fine-tuned on any one category of clinical notes (figure C). The model fine-tuned on neurology notes outperformed each of the diagnostics, EEG, brain MRI, and head CT reports, as well as the full discharge summaries. Finally, comparison of fine-tuning and evaluation of the composite label versus individual labels (eg, drug classes) did not result in a major variation in prediction performance, but it is possible the composite label reduced noise by aggregating each of the categories and as a result best represents the intended task (figure D).

Because methods to interpret large language models are still evolving and do not yet offer clear inter-pretability,^60^ we sought instead to empirically characterise differences between the base clinical-longformer model and the Boston Children’s Hospital clinical-longformer model (table 1). To do this, we explored epilepsy-related prompts for masked language modelling. Specifically, we asked the transformer model to fill in a masked word in a given sentence. Although anecdotal, we observed that the Boston Children’s Hospital model predicted more realistic concepts with a stronger relationship to epilepsy.

## Discussion

These findings yield several important conclusions. Most importantly, we found that physicians encode meaningful prognostic information about seizure recurrence in clinical notes. Although it is difficult for humans to quantify risk as a percentage, these findings indicate that physicians record a sufficient amount of relevant information for large language models to quantify risk with a strong degree of accuracy. Additionally, the improved performance of the models trained on clinical notes shows the utility of leveraging clinical text beyond structured data where feasible. Pre-training large language models on domain-specific (ie, clinical) and sub-domain-specific (ie, paediatric neurological) data can yield substantial improvements. We expect that this trend would also include location-specific data (ie, Boston Children’s Hospital), although demon-strating this was outside the scope of the current study and would require inclusion of additional children’s hospitals. Finally, this study shows that modern EMR-based data warehouses can contain sufficient data for additional pre-training of large language models even for specific tasks (eg, predicting seizure recurrence).

While evaluating this primary task, we identified several additional findings as well as important limitations. We observed that models fine-tuned on neurology notes outperform each other category. Although diagnostic reports tend to report findings in a fact-based manner, progress notes, consult notes, and discharge summaries frequently include subjective sections and even prognoses and future treatment plans. Additionally, we hypothesise that the performance improvement over discharge summaries could be due to two primary factors: patients with discharge summaries were admitted to the hospital at the time of their first seizure-like event as opposed to being seen in an outpatient setting and therefore might be sicker, have additional comorbidities, or other factors that make their prognosis more complicated. This discrepancy could be partly due to the fact that patients are frequently admitted to general paediatrics and then seen by a neurological consultant. This means that the discharge summary is commonly written by a paediatrics resident rather than a paediatric neurologist. The performance difference between discharge summaries and neurology notes could therefore arise because the neurology notes include more specialised insight. An important next step to follow up on these findings would be to develop or use methods as they become available to interrogate large language models to understand what information they are capturing that is not being captured by the structured EMR data.

We observed that children with an encounter for a new-onset seizure-like presentation in the IBM MarketScan database were prescribed an ASM more often than patients in the Boston Children’s Hospital dataset (appendix p 5). A subset of patients at Boston Children’s Hospital might have been treated externally, leading to no prescription being present in the structured EMR data. However, this information was captured in the clinical notes. The remaining difference could have been due to variations in clinical practice (eg, specialised epileptologists at Boston Children’s Hospital might be more adherent to clinical guidelines than general neurologists in the IBM MarketScan cohort, have a higher threshold to start ASMs, keep the possibility of seizure mimics higher in their differential diagnosis, or other reasons). The younger age of patients and lower prevalence of focal epilepsies in the Boston Children’s Hospital cohort could partially explain the lower incidence of ASM prescriptions. For example, simple febrile seizures are fairly common in children aged younger than 5 years, and are relatively clear cases that do not warrant long-term ASM prescription.^11^ Other reasons for differences in clinical practice are not easily testable. Therefore, this is an important limitation to mitigate against when working with EMRs from a single health system, especially if it is a major referral centre for complex cases.

Our comparison of epilepsy-related outcomes within 2 years of an initial seizure-like event illustrates differential effects of biases between a specialised academic health system and a general nationwide cohort. Different modalities of real-world data have distinct strengths and weaknesses for informatics analyses. Large-scale administrative claims datasets present substantial strengths in terms of sample size, as well as the completeness of care recorded across multiple health-care systems and providers. However, administrative claims datasets have a lower resolution of patient health data, and their use for billing and reimbursement or quality reporting and improvement can lead to biases inherent to these processes. By contrast, a single health system’s EMR includes substantially more data per patient, different data types for validation (eg, clinical notes), as well as direct measurements of patient physiology (eg, laboratory and vital measurement results). Despite similar overall rates of seizure recurrence (according to our composite score), fewer patients in the Boston Children’s Hospital cohort had an encounter for status epilepticus. Patients with status epilepticus, a life-threatening event, usually go to the closest emergency department to receive treatment. This might not necessarily have been Boston Children’s Hospital and therefore would not have been captured in the Boston Children’s Hospital EMR. Additionally, more patients at Boston Children’s Hospital received inpatient treatment for non-epilepsy reasons, likely indicating that they had higher medical complexity than patients in the IBM MarketScan dataset. This discrepancy could have influenced health-care provider prescribing habits. These challenges are important to keep in mind when interpreting results based on a single health system.

Throughout this study, we relied on clinical knowledge-based cohort definitions and silver-standard labels in the form of our composite score for seizure recurrence. Both EMRs and administrative claims datasets only capture treatment related to seizures and might not accurately document every seizure. Additionally, paediatric seizures are especially challenging as they often rely on a parent or caregiver observing, recognising, and communicating symptoms to a physician. The composite score was designed to capture seizure recurrence necessitating clinical attention, but it has not been directly validated against a gold-standard prospective trial for capturing recurrent seizures. The best options within routine clinical care would be to examine assets such as long-term video EEG monitoring, but even these would present substantial challenges since these evaluations are typically taken while physicians are adjusting or even withholding medications in order to characterise seizures for successful long-term treatment. Additionally, the increasingly widespread prevalence of smartphones and less restrictive wearables might present the opportunity for improved longitudinal monitoring. It will be worthwhile to explore technologies in this area as they are developed. Although there are limitations to the composite score extracted from real-world data, we believe the stability of prediction models across individual measures helps to illustrate the value of the composite score.

Finally, given the strong performance of the Boston Children’s Hospital clinical-longformer model, there is a compelling reason to further explore this model. First, we intend to replicate these results at additional paediatric hospitals. We expect that models will require substantial pre-training and fine-tuning at each site. Second, we plan to explore prospective assessment where physicians are asked to estimate the recurrence risk for patients matching inclusion criteria. We will assess whether physicians have a strong intuition of seizure recurrence (justifying the 60% threshold set by the International League Against Epilepsy) or whether they are simply capturing sufficient relevant information for a large language model to learn patterns. In conjunction with this, we intend to further explore model interpretation as methods become more robust, to investigate what is driving the predictions. We also intend to observe model performance in more diverse settings, especially where non-specialist physicians are more likely to treat patients after an initial seizure-like event. This approach offers the potential to evaluate whether specialist physicians, in this case child neurologists or epileptologists, or both, hold a more accurate prognostic intuition than non-specialists or general practioners.

We believe there is considerable work to be done in both model development and from an implementation science perspective before making models operational on the basis of clinical notes. The work of Jiang and colleagues^37^ showed the strong performance of large language models in predicting general health-care outcomes in a large health system. Despite the potentially strong performance of such models, however, we have concerns about the clinical deployment of models constructed in this manner. Notably, Jiang and colleagues’^37^ model truncates notes to 512 tokens, whereas in the present study the median number of tokens per note was 1019, meaning their model would cut off nearly half the notes in our dataset. For a note written in the widely used SOAP (subjective, objective, assessment, and plan)^61^ format, this could exclude the entire plan section of the note. The tokeniser used by Jiang and colleagues^37^ might be different and alleviate some of these concerns, but nearly 5% of the notes in our dataset were longer than our context size of 4096. We recognise this as a limitation and urge caution about deploying this model for clinical decision support. Additionally, when looking at the mortality task, within MIMIC-III, approximately 5% of clearly identified admission notes are not finalised before the clinical event being predicted occurs. We therefore do not believe NLP-based approaches are well suited for prediction tasks with short-term outcomes. In our study, the nearest a patient would be considered recurrent is 1 week after an index event. We also urge clarification of the terminology around deployment, since this term has a considerably different meaning in software development and clinical settings. Within software development it might mean simply that the code is running in real time, but in the clinic it typically means that the results are being returned to clinicians and are being considered as part of their decision making. Before clinical deployment of this or other large language models fine-tuned for predictive tasks, a formal study is required, especially as we have substantial concerns about deployment of models on the basis of clinical notes. We are concerned about challenges related to health equity and fairness, the potential for models to “train” physicians to write notes yielding the model prediction they desire (ie, writing notes with a higher likelihood of justifying a more expensive treatment decision), as well as a dataset shift. Despite these limitations, we believe the results shown in this study justify further investigation of how information stored in EMRs could be used to predict the risk of future medical events.

## Supplementary Material

1

## Figures and Tables

**Figure:**
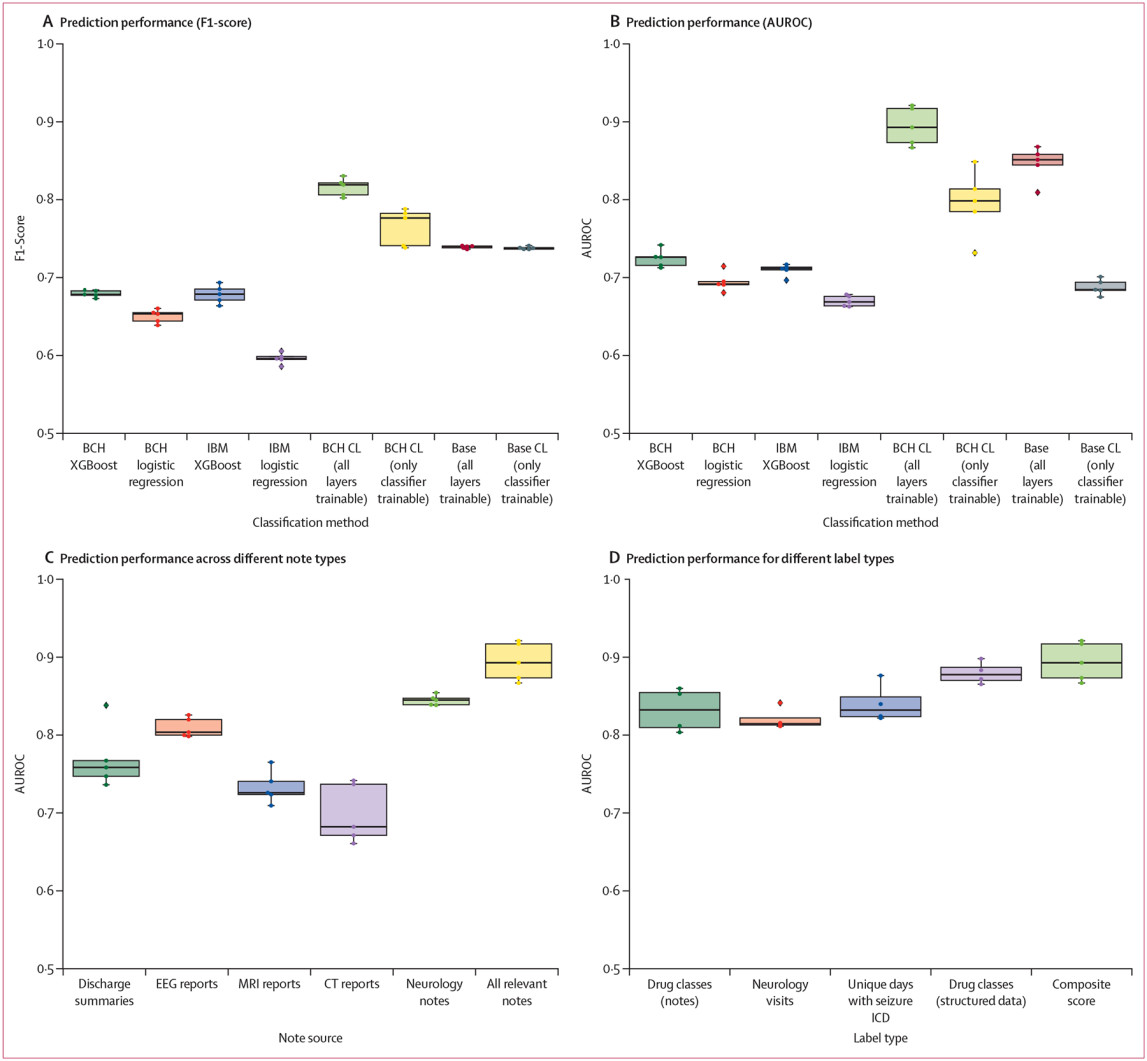
Prediction performance for the composite score of seizure recurrence (A) Comparison of different classifiers by F1-Score. (B) Comparison of different classifiers by area under the receiver operating characteristic (AUROC). (C) Prediction performance providing only specific note types (AUROC). (D) Prediction performance to sub-labels for the composite label (AUROC). Error bars depict 95% CIs. BCH=Boston Children’s Hospital. CL=clinical longformer. EEG=electroencephalogram. ICD=International Classification of Diseases.

**Table 1:** Prompts for understanding the impact of fine-tuning on Boston Children’s Hospital clinical notes

	Base clinical-longformer mask prediction	Boston Children’s Hospital clinical-longformer mask prediction
The patient suffered a <mask>	Stroke, fracture, fall, seizure, trauma	Concussion, fall, seizure, fracture, stroke
The patient experienced a <mask>	Seizure, headache, fall, stroke, fever	Seizure, fall, concussion, headache, fever
The patient’s episode was a <mask>	Brief, trigger, transient, problem, seizure	Seizure, fever, headache, concussion, fall
Patient with refractory <mask>	Hypertension, disease, pneumonia, infection, diabetes	Epilepsy, seizures, seizure, disease, headaches
Patient will undergo elective <mask>	Procedure, surgery, cath, transjugular intrahepatic portosystemic shunt, transesophageal echocardiography	Surgery, admission, MRI, procedure, EEG
The EEG showed <mask>	Normal, improvement, changes, slowing, abnormalities	Seizures, normal, abnormalities, spikes, seizure
Patient experienced a focal <mask>	Headache, seizure, weakness, deficit, pain	Seizure, event, seizures, episode, epilepsy
Leviteracetam is used to treat <mask>	Seizures, this, nausea, pain, anxiety	Seizures, epilepsy, migraine, seizure, depression
Patient has a high likelihood of additional seizures so a <mask> was ordered	EEG, section, transesophageal echocardiography, consult, second	EEG, MRI, PET, CT, video
Patient experienced a <mask> seizure	Generalised, witnessed, second, brief, focal	Breakthrough, generalised, second, prolonged, focal

Top five masked predictions ordered from highest to lowest score. EEG=electroencephalogram.

**Table 2:** Demographics of the study population across Boston Children’s Hospital EMRs and IBM MarketScan dataset

	Boston Children’s Hospital EMR (n=14 021)	IBM MarketScan (n=15 062)	p value
Sex			
Male	7920 (56·5%)*	8013 (53·2%)	>0·25
Female	6100 (43·5%)*	7049 (46·8%)	<0·0001
Age at initial diagnosis (years)	6·76	10·20	<0·0001
<1 year	2228 (15·9%)	622 (4·1%)	<0·0001
1 to <2 years	1566 (11·2%)	1174 (7·8%)	<0·0001
2 to <3 years	768 (5·5%)	1080 (7·2%)	<0·0001
3 to <6 years	2311 (16·5%)	1636 (10·9%)	<0·0001
6 to <11 years	2908 (20·7%)	3127 (20·8%)	<0·0001
11 to <16 years	2367 (16·9%)	3362 (22·3%)	<0·97
16 to <21 years	990 (7·1%)	4061 (27·0%)	<0·0001
Location (state or region)			
Massachusetts	12 196 (87·0%)	··	··
New Hampshire	662 (4·7%)	··	··
Rhode Island	211 (1·5%)	··	··
Maine	198 (1·4%)	··	··
New York	162 (1·2%)	··	··
Connecticut	151 (1·1%)	··	··
No zip code available	150 (1·1%)	··	··
Vermont	43 (0·3%)	··	··
Other	248 (1·8%)	··	··
Northeast	··	2934 (19·5%)	··
North Central	··	3332 (22·1%)	··
South	··	6707 (44·5%)	··
West	··	2089 (13·9%)	··
Race			
White	9535 (68·0%)	NA	··
Other	1726 (12·3%)	NA	··
Black or African-American	1237 (8·8%)	NA	··
Declined to answer	568 (4·1%)	NA	··
Asian	405 (2·9%)	NA	··
Unable to answer	333 (2·4%)	NA	··
Hispanic or Latino	112 (0·8%)	NA	··
Unknown	105 (0·7%)	NA	··
Epilepsy type (most common diagnoses for cases only; table 3)			
Total cases (composite score)	7964	9489	<0·0001
Focal	4050 (50·9%)	5289 (55·7%)	<0·0001
Generalised	3514 (44·1%)	3824 (40·3%)	<0·0001
Even number of focal or generalised	400 (5·0%)	376 (4·0%)	0·0032

Data are n (%). EMR=electronic medical record. NA=not available.

* One patient did not have a recorded value for sex in their medical record. Case definitions for epilepsy diagnosis subgroups are defined in the panel.

**Table 3:** Epilepsy-related outcomes and health-care utilisation in the 2 years after an initial diagnosis

	Median (IQR)		Mean		p value
	Boston Children’s Hospital	IBM MarketScan	Boston Children’s Hospital	IBM MarketScan	
Presence of a diagnosis of status epilepticus*	0 (0–0)	0 (0–0)	0·14 (0·35)	0·25 (0·43)	<0·0001
Number of inpatient admissions for all causes	0 (0–0)	0 (0–0)	1·57 (7·84)	0·91 (2·07)	<0·0001
Number of inpatient admissions for seizures	0 (0–0)	0 (0–0)	0·37 (1·25)	0·34 (0·84)	0·013
General utilisation; number of distinct days with ICD code	7 (3–15)	17 (14–37)	12·82 (19·89)	25·78 (37·78)	<0·0001
Seizure-related utilisation; number of distinct days with seizure-related ICD codes	2 (0–6)	3 (2–10)	4·13 (5·97)	6·40 (14·02)	<0·0001
Number of ASM classes (mean of those receiving at least 1 ASM)	1 (0–2)	1 (0–2)	1·36 (1·79)	1·41 (1·71)	0·17
Number of neurology notes during epilepsy-related ICD visit	3 (1–8)	NA	6·61 (10·90)	NA	NA
Number of seizure-related imaging and procedures	0 (0–1)	0 (0–1)	0·84 (2·35)	0·86 (2·41)	>0·25
Composite score (meeting any of the criteria defined in panel)	1 (0–1)	1 (0–1)	0·57 (0·50)	0·63 (0·48)	<0·001

Data are median (IQR) or mean (SD). ICD=International Classification of Diseases. ASM=antiseizure medication. NA=not available.

* 1=present, 0=absent; mean represents proportion of patients with diagnosis.

## Data Availability

The Boston Children’s Hospital EMR is an identified dataset that requires Boston Children’s Hospital research affiliation and appropriate institutional review board approval to access. Information about accessing the IBM MarketScan research database is available via IBM (https://www.ibm.com/downloads/cas/0NKLE57Y). The source code is available on GitHub (https://github.com/brettbj/seizure-recurrence-prediction) and in archival form on Zenodo (https://doi.org/10.5281/zenodo.8226626).

## Panel: Individual criteria making up the composite score, a heuristic for additional seizures

**Table:**

**Total ASM classes**
Three or more total antiseizure medication (ASM) classes (including rescue therapies; eg, benzodiazepine, see appendix pp 2–3).
**Simultaneous ASM classes**
Two or more ASM classes where each ASM is prescribed at least one additional time after an initial overlapping period.
**Status epilepticus**
Any International Statistical Classification of Diseases (ICD) codes for status epilepticus after initial seizure (ICD-9^44^: 345.2, 345.3, and 345.7; ICD-10^45^: G40.001, G40.011, G40.101, G40.111, G40.201, G40.211, G40.501, G40.801, G40.803, G40.901, G40.911, and G41).
**Inpatient admissions for seizures**
Inpatient admission where epilepsy is listed as primary or admitting billing code (ICD-9 code 345.** or ICD-10 code G40.*; eg, excluding convulsions ICD9–780** and ICD10-R56*).
**Referrals to neurology or neurology notes**
Four or more consult orders or neurology notes, or both, during the course of a visit where epilepsy is listed as a billing code (required to occur on unique days, at least 1 week apart).
**Epilepsy-related procedure orders**
Any of the following procedures during a visit with epilepsy billing codes (at least 1 week after an initial seizure-like event): electroencephalogram, brain MRI, head CT, fMRI, FDG-PET, or surgical treatment (eg, resective epilepsy surgery); the order must occur in an encounter with an epilepsy ICD code and the patient must have at least one more epilepsy ICD code more than 1 week after the procedure.
**Distinct days with epilepsy ICD billing codes**
More than 4 distinct days (each at least 1 week apart) with epilepsy ICD codes (ICD-9 code 345.** or ICD-10 code G40.*).

Patients will be considered to have had additional seizures if they match any of the above criteria in the 2 years after an initial seizure-like event.

## Research in context

### Evidence before this study

We searched PubMed and Google Scholar for publications from database inception to March 7, 2023, using the keywords (“natural language processing” or “deep learning” or “large language models”) AND (“seizure recurrence prediction” or “seizure to epilepsy conversion”) for English language articles. We identified studies that fell into three primary categories: prospective longitudinal clinical studies attempting to estimate seizure recurrence frequency, methods focused on predicting seizure recurrence based on non-text modalities (eg, electroencephalograms), and natural language processing methods to predict general (non-seizure-specific) outcomes from real-world data and clinical notes. To supplement this search, we used UpToDate, an online clinical resource from Wolters Kluwer, to identify synthesised clinical guidelines for the diagnosis, prognosis, and treatment of seizures and epilepsy. These resources provided substantial guidance in determining a composite definition of seizures from real-world data.

### Added value of this study

To date, nearly all approaches based on large language models for prediction with clinical data have focused on broad outcomes (eg, in-hospital mortality and unplanned re-admissions). This retrospective cohort study shows the potential of these large language models in specialist-heavy, narrower tasks. Although it is not possible to conclusively determine whether physicians can accurately predict seizure recurrence with retrospective data in this manner, our findings show that, at a minimum, physicians capture and record a substantial amount of information in the form of clinical notes that can be useful for predicting seizure recurrence in children.

### Implications of all the available evidence

Given that treatment guidelines after an initial seizure-like event rely on a physician’s ability to predict recurrence, this study shows that, at a minimum, physicians know what information is important for this task. Based on these findings, a future prospective study could be conducted whereby physicians are surveyed at the time of an initial seizure-like event and follow-up is carried out with the composite definition for seizure recurrence. Additionally, the strong performance of fine-tuned large language models for narrow, domain-specific tasks could support clinical decision support workflows, but a substantial level of implementation science needs to be done before deployment of such models. These models could also serve as a potential resource to be interrogated for the purposes of developing an aetiological understanding of seizures or examining adherence to clinical guidelines, or both.
