# Supplementary material for: Predicting seizure recurrence after an initial seizure-like episode from routine clinical notes using large language models: a retrospective cohort study
Source: Lancet Digit Health. Author manuscript; Available in PMC 2023 Dec 4. (PMC10695164; doi:10.1016/S2589-7500(23)00179-6)
Supplement: 1 [file NIHMS1947103-supplement-1.pdf]

THE LANCET
Digital Health

Supplementary appendix

This appendix formed part of the original submission and has been peer reviewed. We post it as supplied by the authors.

Supplement to: Beaulieu-Jones BK, Villamar MF, Scordis P, et al. Predicting seizure recurrence after an initial seizure-like episode from routine clinical notes using large language models: a retrospective cohort study. *Lancet Digit Health* 2023; **5:** e882–94.

Supplemental Materials

Supplemental Table 1. Included ICD codes for initial seizure-like encounter.

Epilepsy	ICD-9	345, 345.01, 345.1, 345.11, 345.2, 345.3, 345.4, 345.41, 345.5, 345.51, 345.6, 345.61, 345.7, 345.71, 345.8, 345.81, 345.9, 345.90, 345.91, 780.3, 780.31, 780.32, 780.33, 780.39
	ICD-10	G40.0, G40.00, G40.001, G40.009, G40.01, G40.011, G40.019, G40.1, G40.101, G40.109, G40.111, G40.119, G40.2, G40.20, G40.201, G40.209, G40.21, G40.211, G40.219, G40.3, G40.301, G40.309, G40.311, G40.319, G40.4, G40.401, G40.409, G40.411, G40.419, G40.5, G40.50, G40.501, G40.509, G40.801, G40.802, G40.803, G40.804, G40.811, G40.812, G40.813, G40.814, G40.82, G40.821, G40.822, G40.823, G40.824, G40.89, G40.9, G40.901, G40.909, G40.911, G40.919, G40.A, G40.A0, G40.A01, G40.A09, G40.A1, G40.A11, G40.A19, G40.B, G40.B0, G40.B01, G40.B09, G40.B1, G40.B11, G40.B19, R56, R56.0, R56.00, R56.01, R56.1, R56.9
Focal Epilepsy	ICD-9	345.4, 345.5
	ICD-10	G40.0, G40.00, G40.001, G40.009, G40.01, G40.011, G40.019, G40.1, G40.101, G40.109, G40.111, G40.119, G40.2, G40.20, G40.201, G40.209, G40.21, G40.211, G40.219

Supplemental Table 2. Included and excluded initial antiseizure therapies.[1]

Class	Mechanism	Included as Initial Therapy*	Indication Age
Benzodiazepines (clobazam, clonazepam, clorazepate, diazepam, lorazepam)	GABA Inhibitor	N (adjunctive indications only)	
Brivaracetam	SV2A binding	Y (indicated but not typically used for first line treatment)	4 years[3]
Cannabidiol	Inhibitory transmission	Y (only for Lennox-Gastaut, Dravet, tuberous sclerosis complex)	1-year[3]
Carbamazepine	Sodium channel blocker	Y[2]	
Cenobamate	Sodium channel blocker, GABA-A modulator	N[3]	18 years[3]
Eslicarbazepine	Sodium channel blocker	N (approved for monotherapy but tested in adults for adjunctive therapy)[4,5]	4 years (trial inclusion[3] 16)
Ethosuximide	Calcium channel blocker	Y[6]	2.5 years[3]
Felbamate	NMDA channel blocker, GABA-A modulator[7]	N (not recommended for first line)[7]	
Gabapentin	Calcium channel blocker	Y (primarily studied as adjunctive therapy, but significant usage for first line treatment observed in both datasets)[8]	
Lacosamide	Sodium channel blocker	Y (but frequently used as adjunctive therapy)[9]	4 years, but studied in 1-year[3] 1 year
Lamotrigine	Sodium channel blocker (and other not completely understood mechanisms)	Y (typically adjunctive therapy, but significant support for first line treatment in focal epilepsy)[10–12]	2 years[3]
Levetiracetam	SV2A binding	Y	All
Oxcarbazepine	Sodium channel blocker, increase potassium conductance, Calcium channel blocker	Y[13]	4 years,[3] (adjunctive)
Perampanel	Glutamate receptor antagonist	Y (indicated but not typically used for first line treatment)	4 years[3]
Phenobarbital	GABA-A modulator	Y (particularly neonates)	
Phenytoin	Sodium channel blocker	Y	
Pregabalin	Calcium channel blocker	N (adjunctive)	
Primidone	GABA-A modulator	Y (indicated but not typically used for first line treatment)	
Rufinamide	Sodium channel blocker	N (adjunctive)	
Stiripentol	GABA-A modulator	N (adjunctive, Dravet syndrome)	2 years[3]

Tiagabine	GABA-A modulator	N (adjunctive, focal seizures)	12 years[3]
Topiramate	GABA-A modulator, NMDA-glutamate receptor antagonist	Y	10 years,[3] 2 years (adjunctive)
Valproate	GABA-A modulator, Calcium channel blocker	Y (especially idiopathic generalized epilepsy)	
Vigabatrin	GABA-T inhibitor	Y	
Zonisamide	Sodium and Calcium channel blocker	N (adjunctive but observational support for monotherapy)	

*We included all medications approved for initial therapy even if in practice they are not used as first-line agents (e.g., brivaracetam and cannabidiol are not typically covered by insurance as a first-line treatment but were included as a first-line treatment in prior studies or indications).

Supplemental Table 3. First-line treatment outcome definitions and availability within included data sources.

Outcome	Definition	BCH EMR	IM Claims
Medication Addition	We defined an ASM regimen change to include the addition of an ASM not previously seen before. This allows for when a ASM is switched, or an additional ASM is prescribed. Dosage changes were not examined.<br>Within the BCH EMR, additions included any prescribed therapeutic not previously seen before.<br>Within the IBM Claims data, an addition was defined as the first fill of an ASM in the greater of *a)* 90 days, or *b)* 1.5 times the fill day supply. This definition was designed to detect medication additions where complete pharmaceutical data is available (i.e., in claims) for either 30- and 90-day prescriptions.	X	X
Diagnosis for Status Epilepticus	ICD-9[14]: 345.2, 345.3, 345.7;<br>ICD-10[15]: G40.001, G40.011, G40.101, G40.111, G40.201, G40.211, G40.501, G40.801, G40.803, G40.901, G40.911, G41	X	X
Inpatient Admission for Seizures	DRG groups 100 and 101	X	X
Total Utilization	Count of unique days with an encounter	X	X
Seizure-related Encounters (including unspecified convulsions)	ICD9: 345.*, 780.3*<br>ICD10: G40.*, R56.*	X	X
Epilepsy-related procedures	Imaging, EEG, Implantation/Stimulation Procedures & Resective Surgeries	X	X

Supplemental Table 4. Description of structured data prediction pipeline parameter search.

**Pre-processing**	
Feature Set (10 combinations)	Demographics<br>1. Age at index event<br>2. Biological Sex<br>Diagnoses (ICD was compared to PheCodes)<br>1. Cross-walked to ICD9-CM<br>2. PheCodes[16]<br>Labs<br>1. Loinc codes<br>Procedures (Both codes were included)<br>1. Current Procedure Terminology (CPT)[17]<br>2. ICD-PCS<br>Medications<br>1. NDC to RxNorm Groupings[18,19]<br><br>Combinations:<br>1. Demographics + Diagnoses (ICD)<br>2. Demographics + Diagnoses (PheCodes)<br>3. Demographics + Diagnoses (ICD) + Labs<br>4. Demographics + Diagnoses (PheCodes) + Labs<br>5. Demographics + Diagnoses (ICD) + Medications<br>6. Demographics + Diagnoses (PheCodes) + Medicatoins<br>7. Demographics + Diagnoses (ICD) + Labs + Medications<br>8. Demographics + Diagnoses (PheCodes) + Labs + Medicatoins<br>9. All (ICD)<br>10. All (PheCodes)
Normalization (5 combinations)	Multiple Normalization strategies were compared:<br><br>1. Binary – presence or absence of each code in the year prior to index event<br>2. Count – count of each code in the year prior to<br>3. Log-normalized count (Log of count + 1 clipping the output to [0-1])<br>4. StandardScaler (Scikit-learn)[20]<br>5. MinMaxScaler (Scikit-learn)[20]
Feature Selection (15 combinations)	1. All Features<br>2. All Features present in 1% of training set<br>3. Remove features with low variance (VarianceThreshold)[20]<br>4. Recursive Feature Elimination (K = 50, 100, 500, 1000)<br>5. Top K Features (K=50, 100, 500, 1000) according to:<br>a. Chi-squared statistic

<table>
<tr><td></td><td>b. Mutual Information statistic</td></tr>
<tr><td>Classifier Settings

XGBoost (768 combinations in sweep)

Logistic Regression (120 combinations in sweep)</td><td>XGBoost[21]
1. Booster: gbtree
2. N Estimators: 100, 500, 1000
3. Gamma: 0, 0.1, 0.2
4. Max_depth: 6, 12
5. Sampling_method: ‘uniform’, ‘gradient_based’
6. Lambda (l2 regularization) – 0, 0.1, 1, 2
7. Alpha (l1 regularization) – 0, 0.1, 1, 2

Logistic Regression:
1. Penalty: l1, l2, elasticnet, none
2. C: 0.001, 0.01, 0.1, 1, 10
3. Solver: Liblinear, saga
4. Max Iterations: 100, 500, 1000
5. Class weight: balanced, none</td></tr>
<tr><td>Full Sweep</td><td>XGBoost Grid Search = 576,000 combinations
Results shown from top performing pipeline:
- Feature Set: All (PheCodes)
- Normalization: Log-normalized count
- Feature Selection: All features present in at least 1% of training set (2115 total features)
- Classifier:
o N Estimators: 500
o Gamma: 0
o Max depth: 6 (6 & 12 were not separable)
o Sampling: uniform (no benefit to gradient based)
o Lambda: 0.1
o Alpha: 0.1

Logistic Regression Grid Search = 90,000 combinations
- Feature Set: All (PheCodes)
- Normalization: Log-normalized count
- Feature Selection: Top K – chi-squared (500)
- Penalty: elasticnet (l1 ratio 0.5)
- C: 1
- Max iterations: 500
- Class weight: balanced</td></tr>
</table>

Supplemental Table 5. Breakdown of initial therapy after first seizure-like event (excluding rescue therapy and off label treatment).

	IBM MarketScan	Boston Children's EMR Prescriptions	Boston Children's Medication NLP Extraction
Cohort Size	15,062 (100.00%)	14,021 (100.0%)	14,021 (100.0%)
No first line ASM prescription recorded	3,810 (25.30%)	7,810 (55.70%)	5,694 (40.61%)
Levetiracetam	6,332 (42.04%)	2,737 (19.52%)	3,127 (22.3%)
Oxcarbazepine	1,984 (13.17%)	1,158 (8.26%)	1,424 (10.16%)
Lamotrigine	1,602 (10.64%)	906 (6.46%)	1,001 (7.14%)
Phenobarbital	343 (2.28%)	471 (3.36%)	521 (3.72%)
Gabapentin	292 (1.94%)	419 (2.99%)	404 (2.88%)
Carbamazepine	301 (2.00%)	209 (1.49%)	829 (5.91%)
Lacosamide	115 (0.76%)	122 (0.87%)	789 (5.63%)
Vigabatrin	73 (0.48%)	97 (0.69%)	135 (0.96%)
Phenytoin	190 (1.26%)	76 (0.54%)	83 (0.59%)
Perampanel	14 (0.09%)	8 (0.06%)	5 (0.04%)
Primidone	6 (0.04%)	8 (0.06%)	9 (0.06%)

Supplemental Table 6 Analysis of patients receiving Gabapentin as their first ASM after a seizure-like event (BCH).

	General Cohort	First ASM-Gabapentin	p-value
**Cohort Size**	14,021	419	
**Cases (composite score)**	7,964 (56.8%)	271 (64.7%)	0.11
**Type of Epilepsy**			
Focal	4,050 (50.9%)	184 (67.8%)	< **0.0001**
Generalized	3,514 (44.1%)	77 (28.4%)	< **0.0001**
Even # of focal/generalized	400 (5.0%)	10 (3.8%)	0.69
**Diagnoses during study period**			
Neuropathy	81 (0.6%)	17 (4.1%)	< **0.0001**
Bipolar	166 (1.2%)	9 (2.1%)	0.13
Anxiety	930 (6.6%)	67 (16.0%)	< **0.0001**
Pain	1441 (10.3%)	125 (29.8%)	< **0.0001**
Migraine	830 (5.9%)	70 (16.7%)	< **0.0001**
Headaches (exc. migraines)	1305 (9.3%)	88 (21.0%)	< **0.0001**
Depression	385 (2.7%)	29 (6.9%)	< **0.0001**
Infantile Cerebral Palsy	1713 (12.2%)	115 (27.4%)	< **0.0001**
Encephalopathy	808 (5.8%)	53 (12.6%)	< **0.0001**

Supplemental Table 7. Prediction performance and 95% confidence intervals corresponding with Figure 1A and 1B.

**Method**	**F1-Score:** mean [95% CI]	**AUROC:** mean [95% CI]
BCH XGBoost	0.679 [0.676 - 0.683]	0.725 [0.717 - 0.734]
BCH Logistic Regression	0.650 [0.643 - 0.657]	0.694 [0.685 - 0.705]
IBM XGBoost	0.678 [0.668 - 0.687]	0.710 [0.703 - 0.714]
IBM Logistic Regression	0.596 [0.590 - 0.601]	0.670 [0.664 - 0.675]
BCH CL (All layers trainable)	0.826 [0.817 - 0.835]	0.897 [0.875 - 0.913]
BCH CL (Only classifier trainable)	0.765 [0.748 - 0.784]	0.795 [0.759 - 0.829]
Base CL (All Layers Trainable)	0.739 [0.738 - 0.741]	0.846 [0.826 - 0.861]
Base CL (Only classifier trainable)	0.738 [0.737 - 0.740]	0.688 [0.680 - 0.696]

Supplemental Table 8. Study cohort geographic locations with and without requirements for non-epilepsy related encounters.

	**Study Cohort**	**Study Cohort without baseline encounter requirement**
**Massachusetts**	12,196 (87.0%)	25,180 (70.1%)
**New Hampshire**	662 (4.7%)	2,081 (5.8%)
**Rhode Island**	211 (1.5%)	852 (2.4%)
**Maine**	198 (1.4%)	814 (2.3%)
**New York**	162 (1.2%)	780 (2.2%)
**Connecticut**	151 (1.1%)	728 (2.0%)
**No Zip Code Available**	150 (1.1%)	1,165 (3.2%)
**Florida**	51 (0.4%)	276 (0.8%)
**Vermont**	43 (0.3%)	217 (0.6%)
**Other States**	197 (1.4%)	4,028 (11.2%)
**Total**	**14,021**	**35,898**
**New England**	13,461 (96.0%)	29,872 (83.2%)

Supplemental References

1 Schachter SC. Antiseizure medications: Mechanism of action, pharmacology, and adverse effects. In: Garcia P, Dashe JF, eds. UpToDate. Waltham, MA: UpToDate, 2021.

2 Glauser T, Ben-Menachem E, Bourgeois B, *et al.* Updated ILAE evidence review of antiepileptic drug efficacy and effectiveness as initial monotherapy for epileptic seizures and syndromes. *Epilepsia* 2013; **54**: 551–63.

3 Office of the Commissioner. FDA approves new treatment for adults with partial-onset seizures. 2019. https://www.fda.gov/news-events/press-announcements/fda-approves-new-treatment-adults-partial-onset-seizures (accessed June 15, 2021).

4 Chang X, Yuan H, Wang Y, Xu H, Hong W, Zheng R. Eslicarbazepine acetate add-on for drug-resistant focal epilepsy. *Cochrane Database Syst Rev* 2017. DOI:10.1002/14651858.CD008907.pub3.

5 Sperling MR, Abou-Khalil B, Harvey J, *et al.* Eslicarbazepine acetate as adjunctive therapy in patients with uncontrolled partial-onset seizures: Results of a phase III, double-blind, randomized, placebo-controlled trial. *Epilepsia* 2015; **56**: 244–53.

6 Glauser TA, Cnaan A, Shinnar S, *et al.* Ethosuximide, valproic acid, and lamotrigine in childhood absence epilepsy. *N Engl J Med* 2010; **362**: 790–9.

7 Troupin AS, Montoris G, Hussein G. Felbamate: Therapeutic range and other kinetic information. *J Epilepsy* 1997; **10**: 26–31.

8 Marson AG, Kadir ZA, Hutton JL, Chadwick DW. Gabapentin add-on for drug-resistant partial epilepsy. *Cochrane Database Syst Rev* 2000; : CD001415.

9 Guilhoto LMFF, Loddenkemper T, Gooty VD, *et al.* Experience with lacosamide in a series of children with drug-resistant focal epilepsy. *Pediatr Neurol* 2011; **44**: 414–9.

10 Biton V, Di Memmo J, Shukla R, *et al.* Adjunctive lamotrigine XR for primary generalized tonic-clonic seizures in a randomized, placebo-controlled study. *Epilepsy Behav* 2010; **19**: 352–8.

11 Nevitt SJ, Sudell M, Cividini S, Marson AG, Tudur Smith C. Antiepileptic drug monotherapy for epilepsy: a network meta-analysis of individual participant data. *Cochrane Database Syst Rev* 2022; **4**: CD011412.

12 Marson AG, Burnside G, Appleton R, *et al.* Lamotrigine versus levetiracetam or zonisamide for focal epilepsy and valproate versus levetiracetam for generalised and unclassified epilepsy: two SANAD II non-inferiority RCTs. *Health Technol Assess* 2021; **25**: 1–134.

13 Koch MW, Polman SK. Oxcarbazepine versus carbamazepine monotherapy for partial onset seizures. *Cochrane Database Syst Rev* 2009; : CD006453.

14 Organization WH, Others. International classification of diseases:[9th] ninth revision, basic tabulation list with alphabetic index. 1978. https://apps.who.int/iris/handle/10665/39473.

15 Brämer GR. International statistical classification of diseases and related health problems. Tenth revision. *World Health Stat Q* 1988; **41**: 32–6.

16 Bastarache L. Using Phecodes for Research with the Electronic Health Record: From PheWAS to PheRS. *Annu Rev Biomed Data Sci* 2021; **4**: 1–19.

17 American Medical Association. Cpt 1999: Current Procedural Terminology. Wolters Kluwer Law & Business, 1995.

18 Liu S, Ma W, Moore R, Ganesan V, Nelson S. RxNorm: prescription for electronic drug information exchange. *IT Prof* 2005; **7**: 17–23.

19 Nelson SJ, Zeng K, Kilbourne J, Powell T, Moore R. Normalized names for clinical drugs: RxNorm at 6 years. *J Am Med Inform Assoc* 2011; **18**: 441–8.

20 Pedregosa F, Varoquaux G, Gramfort A. Scikit-learn: Machine learning in Python. *J Mach Learn Res* 2011. http://www.jmlr.org/papers/v12/pedregosa11a.html.

21 Chen, He, Benesty, Khotilovich. Xgboost: extreme gradient boosting. *R package version* 2015. https://cran.microsoft.com/snapshot/2017-12-11/web/packages/xgboost/vignettes/xgboost.pdf.
